# Author Correction: A single-cell atlas and lineage analysis of the adult *Drosophila* ovary

**DOI:** 10.1038/s41467-021-26191-1

**Published:** 2021-10-06

**Authors:** Katja Rust, Lauren E. Byrnes, Kevin Shengyang Yu, Jason S. Park, Julie B. Sneddon, Aaron D. Tward, Todd G. Nystul

**Affiliations:** 1 UCSF, Department of Anatomy, 513 Parnassus Ave, San Francisco, CA 94143 USA; 2 UCSF, Department of OB-GYN/RS, 513 Parnassus Ave, San Francisco, CA 94143 USA; 3 Broad Center of Regeneration Medicine and Stem Cell Research, 513 Parnassus Ave, San Francisco, CA 94143 USA; 4 UCSF, Department of Cell and Tissue Biology, 513 Parnassus Ave, San Francisco, CA 94143 USA; 5 UCSF, Department of Otolaryngology-Head and Neck Surgery, 513 Parnassus Ave, San Francisco, CA 94143 USA; 6 UCSF, Diabetes Center, 513 Parnassus Ave, San Francisco, CA 94143 USA

**Keywords:** RNA sequencing, Developmental biology, Genetics

Correction to: *Nature Communications* 10.1038/s41467-020-19361-0, published online 6 November 2020.

This Article contained an error in the spelling of the author Lauren E. Byrnes, which was incorrectly given as Laurean E. Byrnes.

In addition, the original version of this Article contained an error in Fig. 3P and Fig. 6B. In the original version of Fig. 3P, the data for 7 dpts and 14 dpts were inadvertently inverted for both genotypes.

The correct version of this panel is:
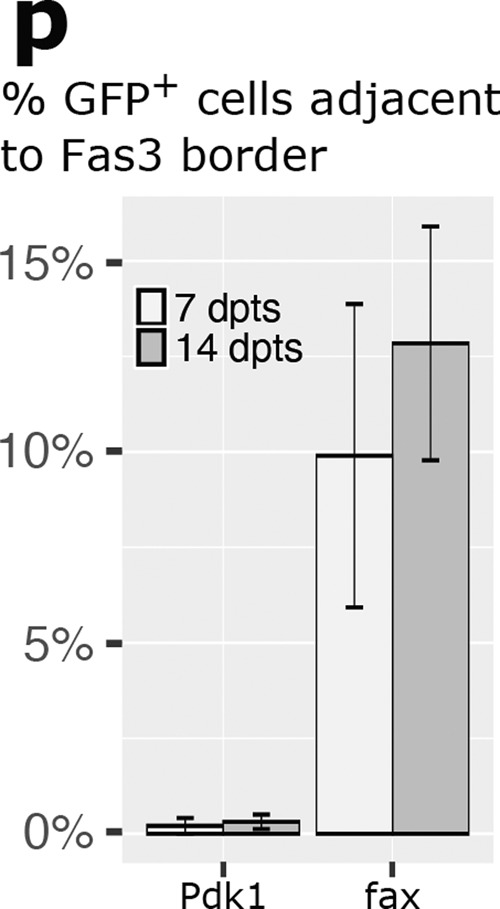


Which replaces
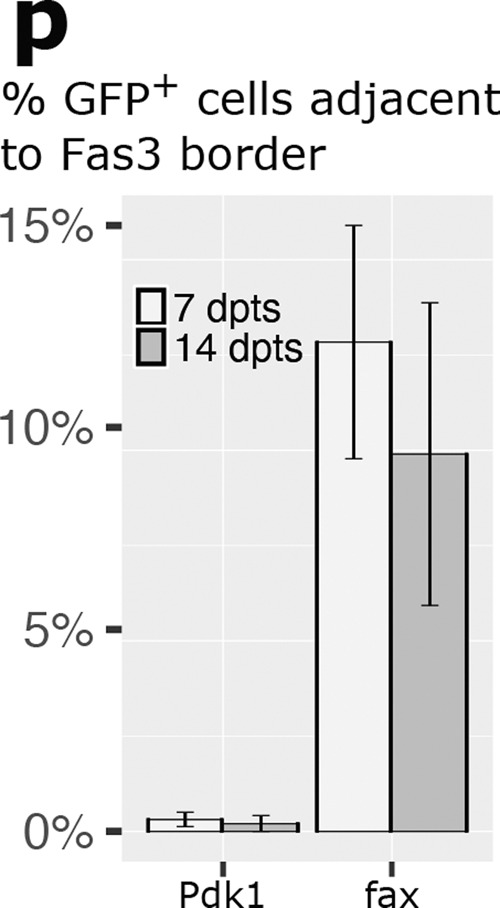


In the original version of Figure 6B, the range of colours used to indicate correlation in gene expression between different cell types were inadvertently inverted.

The correct version of this panel is:
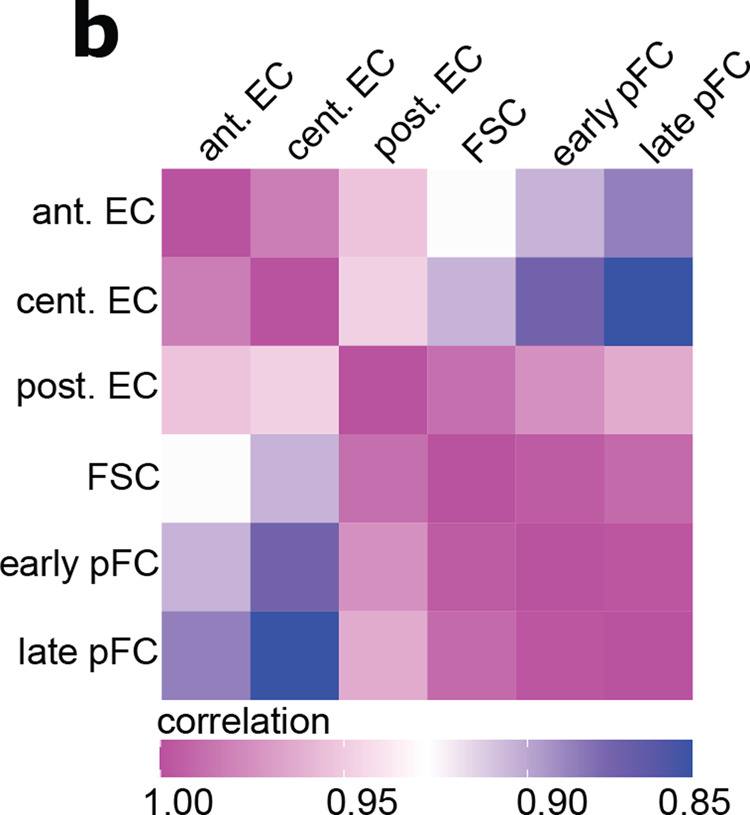


Which replaces
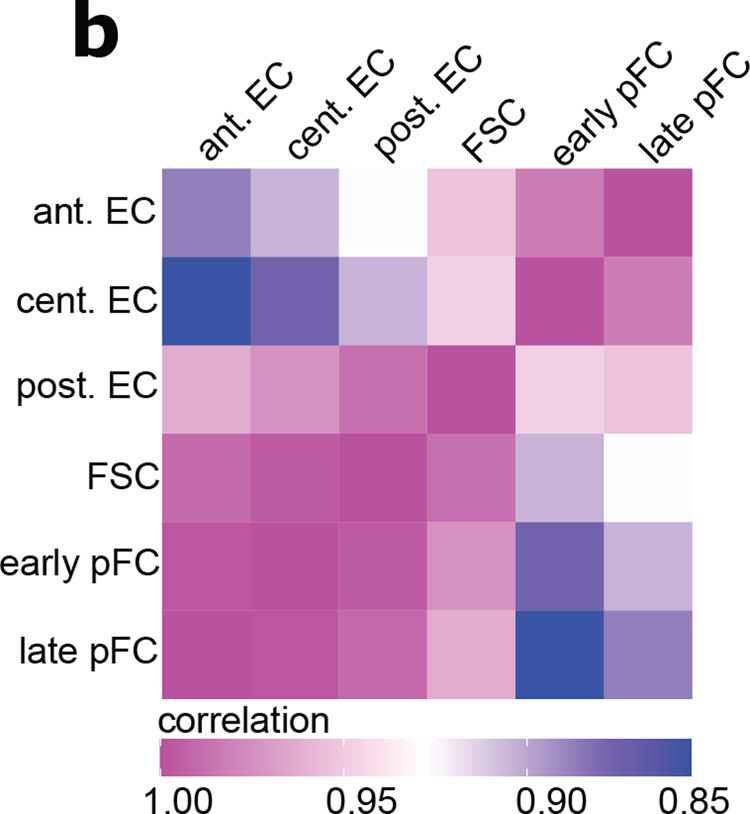


Furthermore, the original version of this Article contained errors in the Introduction and Discussion, which omitted to discuss available data regarding the location of FSCs, especially relative to the boundary of Fas3 expression. In the first paragraph of the Introduction after the fifth sentence, the following sentence has been added “The FSCs are known to reside at the anterior edge of the follicle epithelium, but the precise number and location of FSCs within the germarium has been debated recently. In particular, whereas our recent study supports the view that FSCs have low but detectable levels of the follicle cell marker, Fas3, and are thus on the posterior side of the Fas3 expression boundary^3,4^, other studies suggest that FSCs are Fas3– and are thus anterior to the Fas3 expression boundary^5–7^”.

In addition, in the third paragraph of the Discussion, the first sentence “Our use of G-TRACE to assess the lineage potential of somatic cells in the germarium led to the surprising finding that the ECs can convert to FSCs under starvation conditions” has been replaced with the following: “Our use of G-TRACE to assess the lineage potential of somatic cells in the germarium generated several insights. First, it provided support for the view that FSCs reside within the Fas3 expression boundary and express low levels of Fas3^3,4^, rather than anterior to the Fas3 expression boundary, as proposed recently^5–7^. Specifically, we found that Wnt4-Gal4 driving G-TRACE produced FSC clones with very high frequency whereas fax-Gal4 driving the same G-TRACE construct typically did not, at least under well-fed conditions. Analysis of RFP expression in these ovarioles indicated that Wnt4-Gal4 is expressed at low levels in the weakly Fas3+ cells at the Fas3 expression boundary whereas fax-Gal4 is not expressed in these cells, suggesting that expression within the Fas3+ population is required to produce FSC clones in well-fed conditions. Second, further analysis of the flies with fax-Gal4 driving G-TRACE led to the surprising finding that ECs can convert to FSCs under starvation conditions”.

The following references have been added to reflect these changes:

3. Fadiga, J. & Nystul, T. G. The follicle epithelium in the *Drosophila* ovary is maintained by a small number of stem cells. *Elife* **8**, (2019).

4. Nystul, T. G. & Spradling, A. Regulation of epithelial stem cell replacement and follicle formation in the Drosophila ovary. *Genetics* **184**, 503–515 (2010).

5. Reilein, A. et al. Alternative direct stem cell derivatives defined by stem cell location and graded Wnt signalling. *Nat. Cell Biol*. **19**, 433–444 (2017).

6. Reilein, A., Melamed, D., Tavaré, S. & Kalderon, D. Division-independent differentiation mandates proliferative competition among stem cells. *Proc. Natl. Acad. Sci. USA* **115**, E3182–E3191 (2018).

7. Melamed, D. & Kalderon, D. Opposing JAK-STAT and Wnt signaling gradients define a stem cell domain by regulating differentiation at two borders. *Elife* **9**, (2020).

References numbers have been updated throughout in light of these additions.

These errors have been corrected in both the PDF and HTML versions of the Article.

