# Supplementary material for: A single-cell atlas and lineage analysis of the adult Drosophila ovary
Source: Nat Commun. 2020 Nov 6;11:5628. doi: 10.1038/s41467-020-19361-0 (PMC7648648; doi:10.1038/s41467-020-19361-0)
Supplement: Supplementary file 1 — Supplementary Information [file 41467_2020_19361_MOESM1_ESM.pdf]

Supplementary Information

A Single-Cell Atlas and Lineage Analysis of the Adult Drosophila Ovary

Katja Rust[1,2,5], Lauren E. Byrnes[1,3], Kevin Shengyang Yu[4], Jason S. Park[4], Julie B. Sneddon[1,3,5,6], Aaron D. Tward[4], Todd G. Nystul[1,2,*]

[1]UCSF, Department of Anatomy, 513 Parnassus Ave, San Francisco, CA, 94143
[2]UCSF, Department of OB-GYN/RS, 513 Parnassus Ave, San Francisco, CA, 94143
[3]UCSF, Department of Cell and Tissue Biology, 513 Parnassus Ave, San Francisco, CA, 94143
[4]UCSF, Department of Otolaryngology-Head and Neck Surgery, 513 Parnassus Ave, San Francisco, CA, 94143
[5]Broad Center of Regeneration Medicine and Stem Cell Research, 513 Parnassus Ave, San Francisco, CA, 94143
[6]UCSF, Diabetes Center, 513 Parnassus Ave, San Francisco, CA, 94143
*Corresponding author: todd.nystul@ucsf.edu

Contents:

Supplementary Figures 1-10
Supplementary Tables 1-3

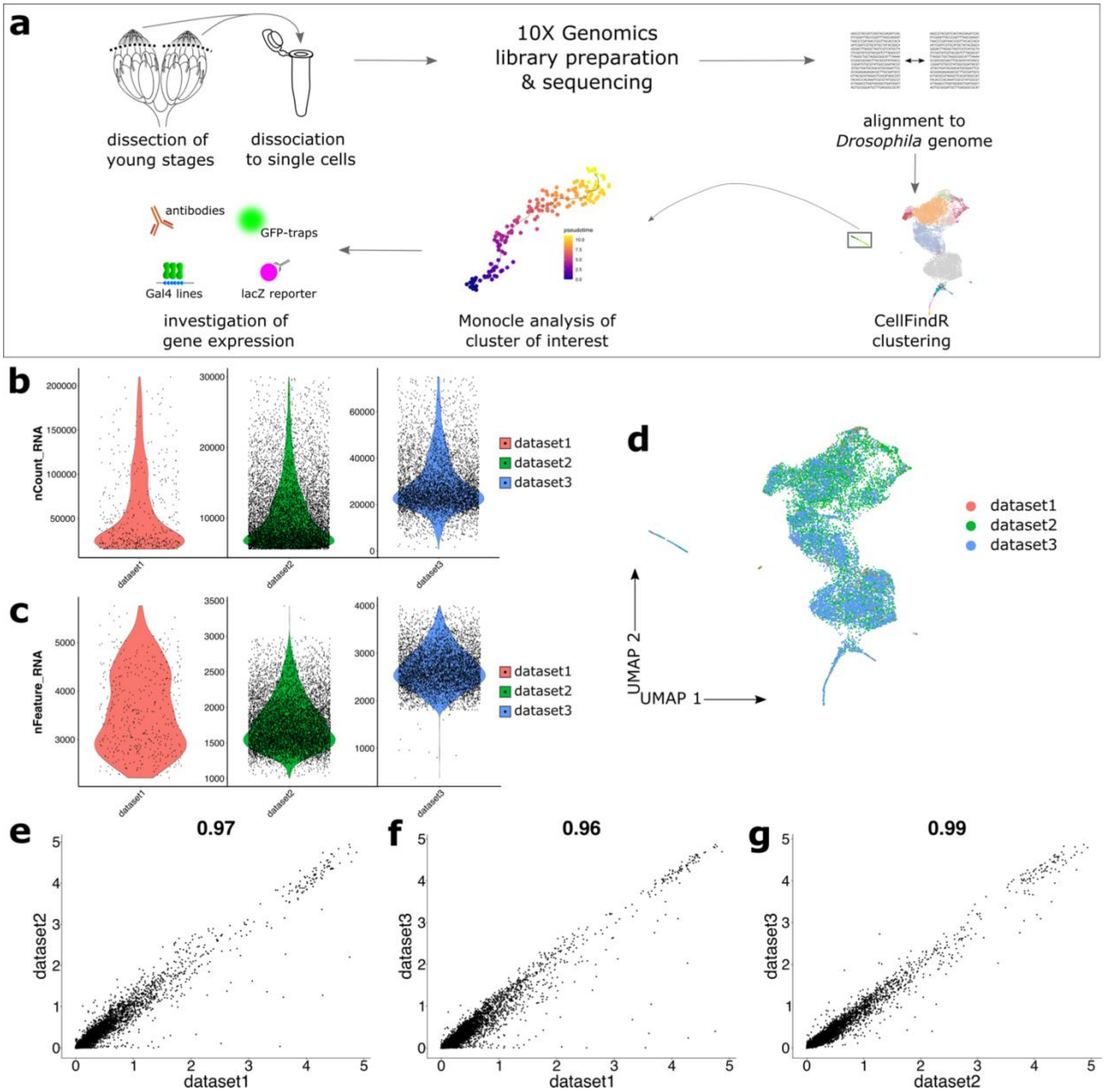

Supplementary Figure 1: scRNA-seq dataset quality and comparison of Seurat and CellFindR

a) Schematic overview of sample preparation for single-cell sequencing. b-c) Violin plots summarizing the number of high quality cells and the filter settings for each dataset. d) UMAP plot showing the distribution of each dataset across each cluster. e-g) Pairwise Pearson correlations of gene expression levels indicates strong correlations between the three datasets.

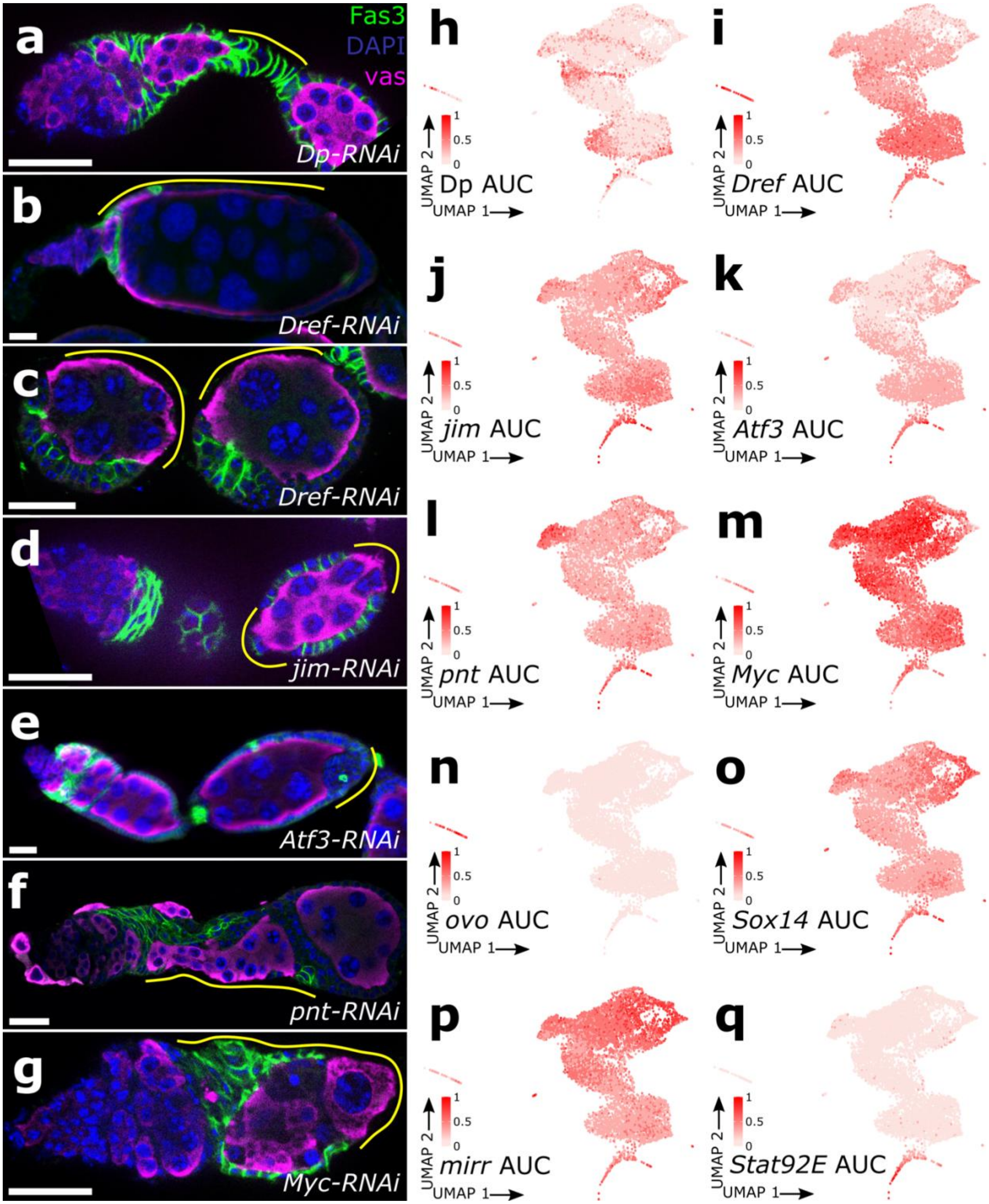

Supplementary Figure 2: SCENIC identifies transcription factors that are required for oogenesis

a-g) To validate predictions from SCENIC, we selected a subset of regulons with different patterns of activity across the dataset and used 109-30-Gal4; tub-Gal80ts to drive expression of the corresponding transcription factor specifically during adulthood in the early follicle cell lineage and stained ovarioles for Fas3 (green),

vasa (magenta), and DAPI (blue). Representative examples of ovarioles with significant morphological defects (yellow line) are shown. Interestingly, although Myc transcript is not detected in the early follicle cell lineage, SCENIC predicts that the Myc regulon is highly active and, indeed, we observe a strong morphological phenotype upon RNAi knockdown of Myc in these cells suggesting that the gene is expressed in these cells but at a level that is too low to be detected in our scRNA-seq dataset. Scale bars = 20 µm. h-m) The pattern of regulon activity corresponding to each of the transcription factors tested in a-g in dataset2. Scale bars show the percent of regulon activity. n-q) Dataset2 UMAP plot examples of additional regulons with patterns of activity that align with expectations. The regulon for ovo, which is required for germ cell survival and differentiation,[1] is active in germ cell clusters (n). Likewise, the regulons for Sox14, which we identify as a novel marker of late-stage follicle cells (Fig. 7), and mirr, which is a marker of central follicle cells in mid-oogenesis are active in the corresponding clusters (p-q). Stat92E regulon activity is high in early follicle cells and stalk cells consistent with the importance of JAK-STAT signaling in these cell types[2,3] (q). Scale bars show the percent of regulon activity. AUC: Area under the curve.

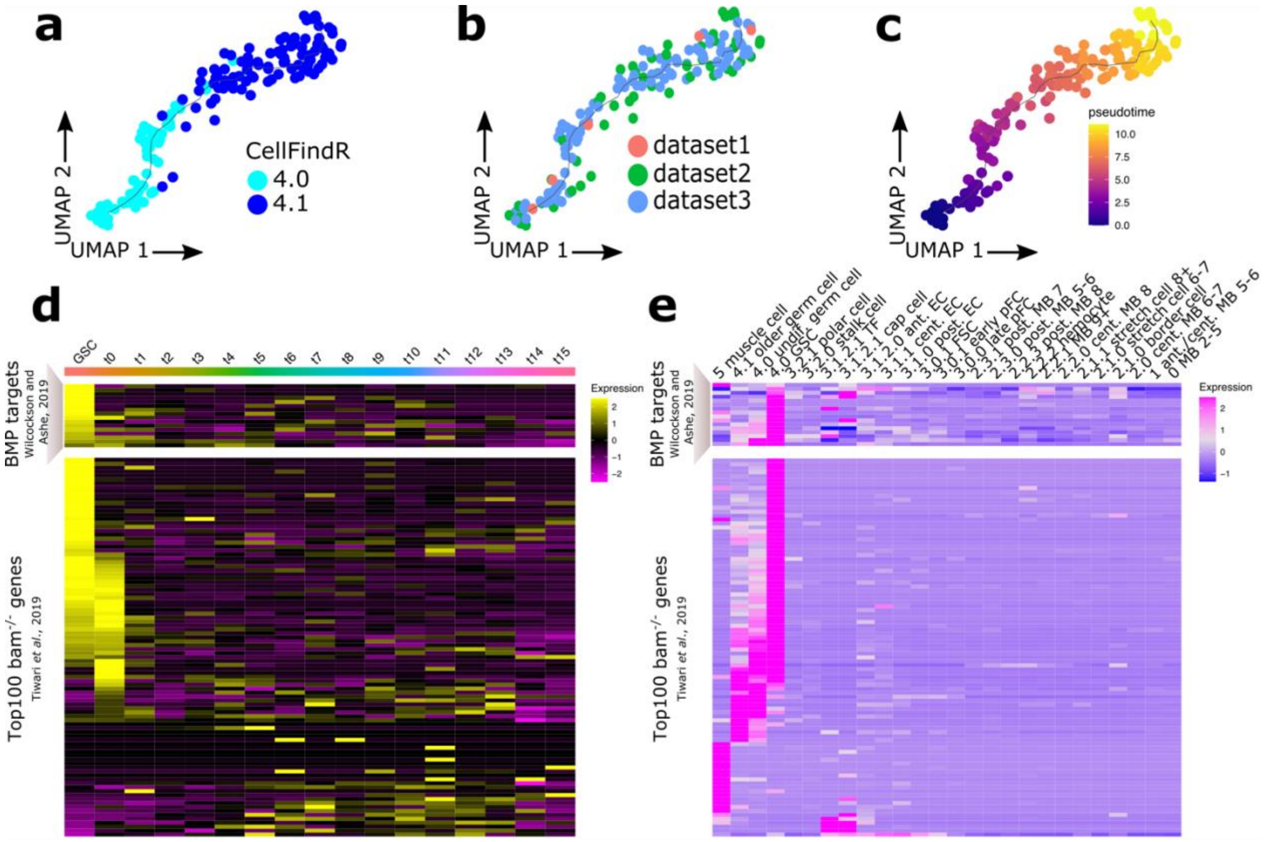

Supplementary Figure 3: Monocle3 analysis of germ cells identifies germline stem cells

a-c) Pseudotime trajectories of germ cells showing the position of cells in each cluster (A), in each dataset (B), and across pseudotime (c). d-e) Heat map showing the expression BMP responsive genes[4] and of the top 100 genes that are most strongly enriched in GSC-like *bam*$^{-/-}$ tumors versus wildtype[5] in each cluster (d) and across pseudotime (e). These genes are strongly enriched in germ cells at the earliest stages of pseudotime, as expected. undif.: undifferentiated; GSC: germline stem cell; TF: terminal filament; EC: escort cell; ant.: anterior; cent.: central; post.: posterior; FSC: follicle stem cell; pFC: prefollicle cell; MB: main body follicle cell.

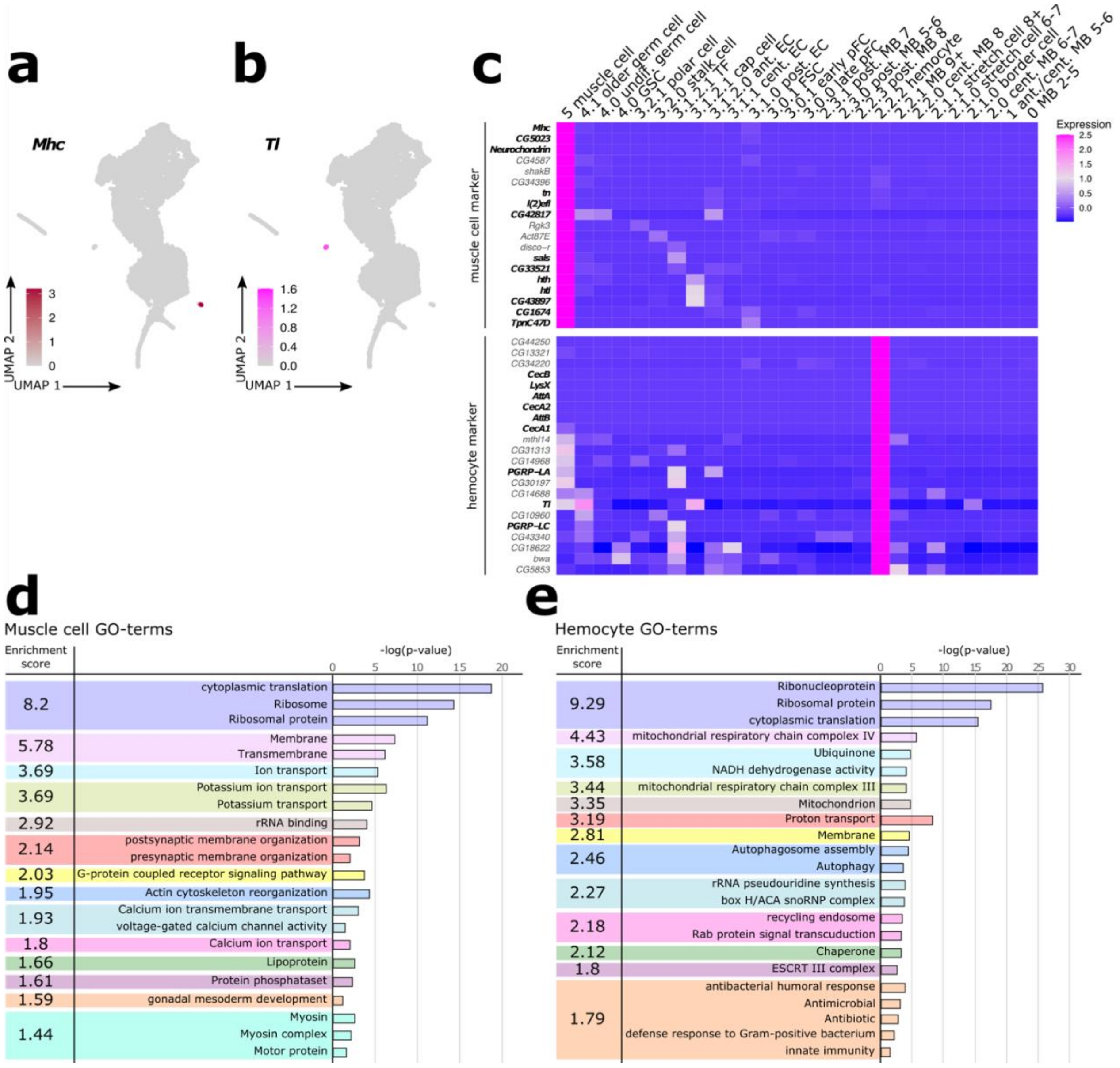

Supplementary Figure 4: Muscle cell and hemocytes

a-b) UMAP plots showing the expression of the muscle cell marker, *Mhc* (A) or the hemocyte marker, *Tl* (b). c) Heat map showing the expression of muscle cell and hemocyte markers across the entire dataset. Bolded gene names indicate genes with a confirmed function in the corresponding cell type; non-bolded gene names are candidate novel markers. d-e) Tables showing the enrichment scores and p-values of GO-terms that are enriched in muscle cells (d) and hemocytes (e). undif.: undifferentiated; GSC: germline stem cell; TF: terminal filament; EC: escort cell; ant.: anterior; cent.: central; post.: posterior; FSC: follicle stem cell; pFC: prefollicle cell; MB: main body follicle cell.

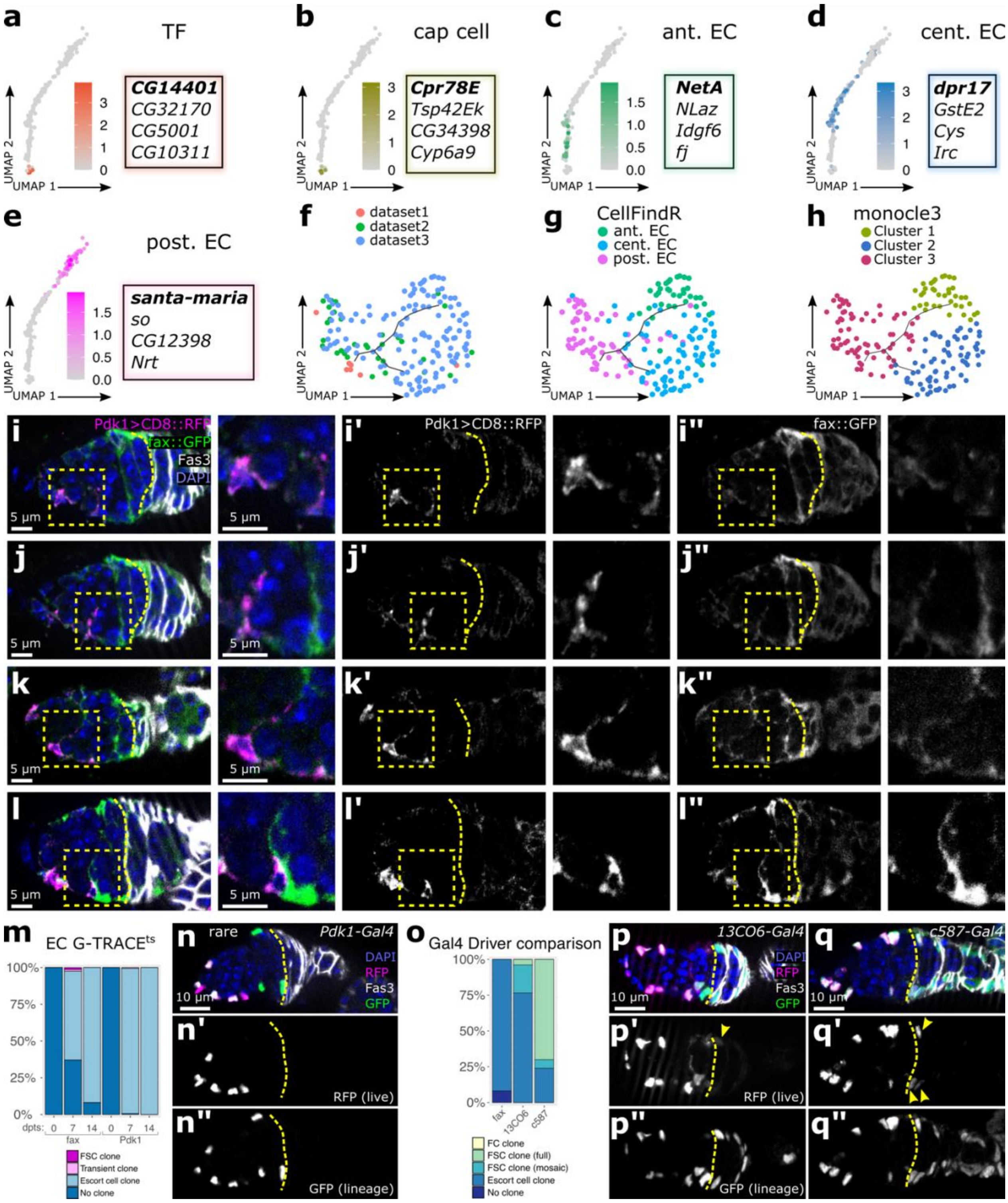
a
TF
CG14401
CG32170
CG5001
CG10311
b
cap cell
Cpr78E
Tsp42Ek
CG34398
Cyp6a9
c
ant. EC
NetA
NLaz
Idgf6
fj
d
cent. EC
dpr17
GstE2
Cys
Irc
e
post. EC
santa-maria
so
CG12398
Nrt
f
dataset1
dataset2
dataset3
g
CellFindR
ant. EC
cent. EC
post. EC
h
monocle3
Cluster 1
Cluster 2
Cluster 3
UMAP 1
UMAP 2
Pdk1>CD8::RFP
fax::GFP
Fas3
DAPI
5 µm
m
EC G-TRACE^ts
dpts:
fax
Pdk1
FSC clone
Transient clone
Escort cell clone
No clone
n
rare
Pdk1-Gal4
RFP
GFP
10 µm
RFP (live)
GFP (lineage)
o
Gal4 Driver comparison
13CO6
c587
FC clone
FSC clone (full)
FSC clone (mosaic)
Escort cell clone
No clone
p
13CO6-Gal4
q
c587-Gal4

Supplementary Figure 5: Markers, monocle3 analysis, and *in vivo* validation of anterior germarial somatic cells

a-e) UMAP plots of anterior germarial somatic cells showing marker expression pattern for each cluster. Marker in bold is shown on plot. Additional markers are listed below. f-h) UMAP plots showing pseudotime trajectory of ECs generated with monocle3. Datasets are distributed across the trajectory (f). CellFindR and monocle3 clusters align well (g-h). i-l) Germaria with *fax::GFP* and *Pdk1-Gal4* driving CD8::RFP stained for RFP (magenta), GFP (green), Fas3 (white), and DAPI (blue). i'-l') CD8::RFP (white). i"-l") fax::GFP (white). *Pdk1-Gal4* is expressed in ECs with short protrusions (i'-l') but not in posterior ECs with long protrusions (i"-l"). Yellow dotted line demarks the Region 2a/2b border. Boxed region is enlarged on the left. m) Quantification of ovarioles without GFP^+ cells (no clone), only GFP^+ ECs (Escort cell clone), or GFP^+ ECs with transient follicle cell clones (Transient clone) or FSC clones (FSC clone) in germaria with *fax-Gal4* or *Pdk1-Gal4* driving G-$TRACE^{ts}$ well-fed for 0, 7, or 14 days before dissection. n = 167, 167, and 170 ovarioles for *fax-Gal4* at 0, 7, and 14 dpts, respectively. 113, 159, 134 ovarioles for *Pdk1-Gal4* at 0, 7, and 14 dpts, respectively. Note that ovarioles with follicle cell clones derived from a single fly. n) Rare germarium with *Pdk1-Gal4* driving G-$TRACE^{ts}$ with GFP^+ cells at the Region 2a/2b border. Germarium is stained for RFP (magenta), GFP (green), Fas3 (white) and DAPI (blue). n') RFP (white). n") GFP (white). Yellow line demarks the Region 2a/2b border. o) Frequencies of each clone type produced by *fax-Gal4*, *13C06-Gal4*, and *c587-Gal4* driving G-$TRACE^{ts}$ at well-fed conditions for 14 days before dissection. n = 170, 51, and 50 ovarioles for *fax-Gal4*, *13C06-Gal4*, and *c587-Gal4*, respectively. p-q) Germaria expressing $GTRACE^{ts}$ under control of *13CO6-Gal4* (p) or *c587-Gal4* (q) stained for RFP (magenta), GFP (green), Fas3 (white) and DAPI (blue). p'-q') RFP staining (white). p"-q") GFP staining (white). Yellow line outlines the 2a/2b border. Arrows point at RFP^+ follicle cells showing expression is not restricted to ECs. TF: terminal filament; EC: escort cell; ant.: anterior; cent.: central; post.: posterior; dpts: days post temperature shift.

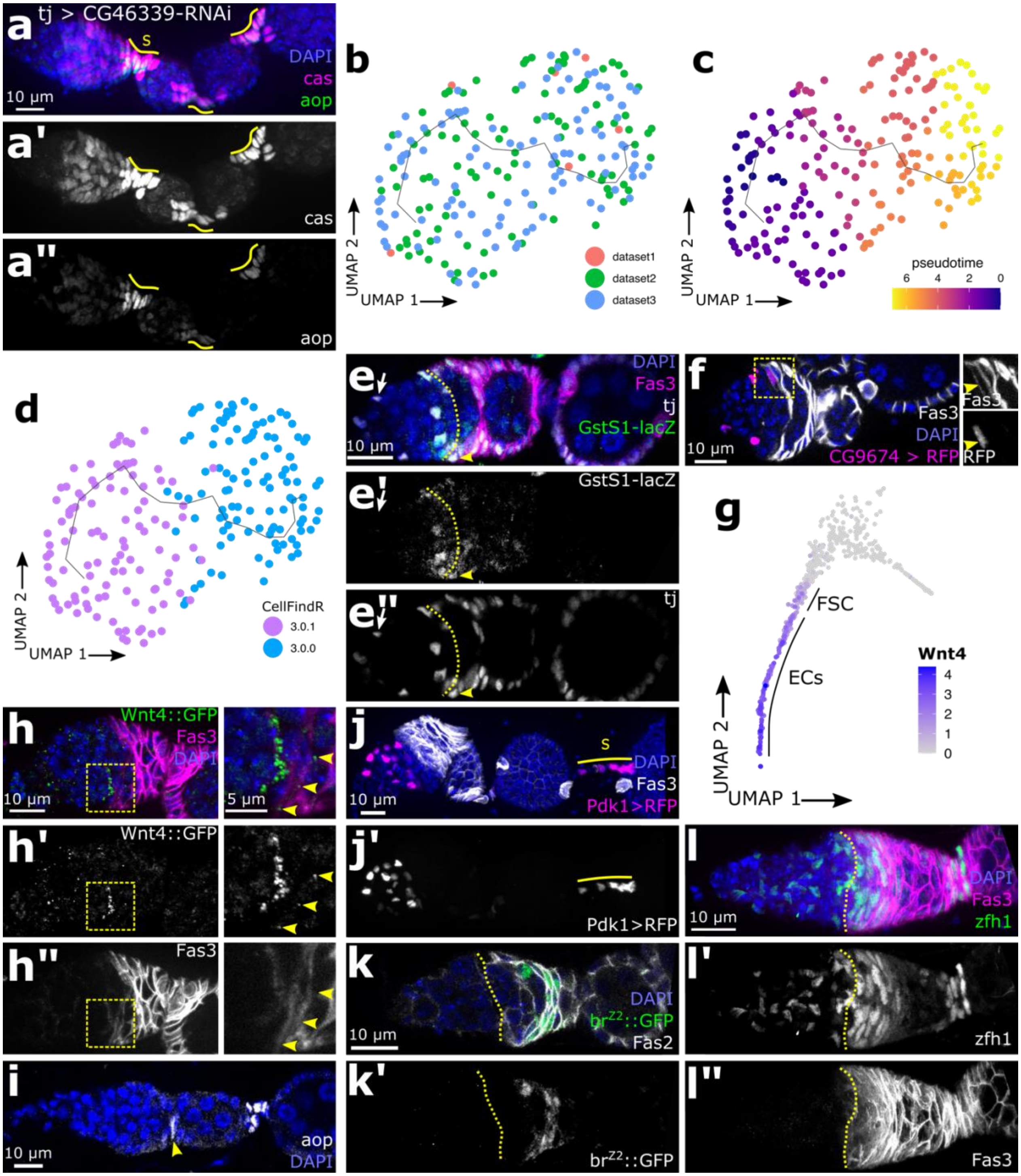

Supplementary Figure 6: Markers of early stages in the FSC lineage

a) Maximum intensity projection of germarium with *tj-Gal4* driving *CG46339-RNAi* stained for cas (magenta), aop (green) and DAPI (blue). a') cas (white). a") aop (white). Stalks form properly and show normal cas and aop expression. b-d) UMAP plots showing follicle cell pseudotime trajectory. Datasets are distributed

across the trajectory (b). monocle3 sorts cells in cluster 3.0.1 containing FSCs and early pFCs at the beginning of pseudotime and cells in cluster 3.0.0 containing late pFCs later in pseudotime (c-d). e) *GstS1-LacZ* germarium stained for LacZ (green), Fas3 (magenta), tj (white) and DAPI (blue). e') GstS1-lacZ (white). e") tj (white). GstS1 is expressed in somatic cells at Region 2a/2b border, including pECs, FSCs (arrowhead), and early pFCs. f) Germarium with *CG9674-Gal4* driving RFP stained for RFP (magenta), Fas3 (white), and DAPI (blue). Insets: magnified view of indicated square in Fas3 and RFP channels (white). Arrowhead: RFP^+, $Fas3^+$ cell. g-h) UMAP plot (g) showing *Wnt4* expression in ECs and FSCs, and *Wnt4::GFP* germarium stained for GFP (green), Fas3 (magenta), and DAPI (blue) (h). h') GFP (white). (h") Fas3 (white). Arrowheads: GFP^+ puncta in Fas3 positive cells at the Fas3 border. i) Wildtype germarium stained for aop (white) and DAPI (blue) confirms aop expression in stalk cells and their precursors (arrowhead). j) Maximum intensity projection of a germarium with *Pdk1-Gal4* driving RFP stained for RFP (magenta), Fas3 (white), and DAPI (blue) showing *Pdk1-Gal4* expression in stalk cells (s). j') RFP (white). k) br^{Z2}*-GFP* germarium stained for GFP (green), Fas3 (magenta), and DAPI (blue) reveals *br* expression in Region 3 pFCs. k') GFP (white). Note that an anti-BrC antibody[6] detecting the entire broad complex does not detect a signal in the germarium. l) Maximum intensity projection of a wildtype germarium stained for zfh-1 (green), Fas3 (magenta), and DAPI (blue). l') zfh1 (white). l") Fas3 (white). zfh-1 is expressed weakly in anterior ECs and central ECs, strongly in posterior ECs, FSCs and prefollicle cells in Region 2b, but is expressed low or not detectable in Region 3. Yellow line: Region 2a/2b border. FSC: follicle stem cell; EC: escort cell.

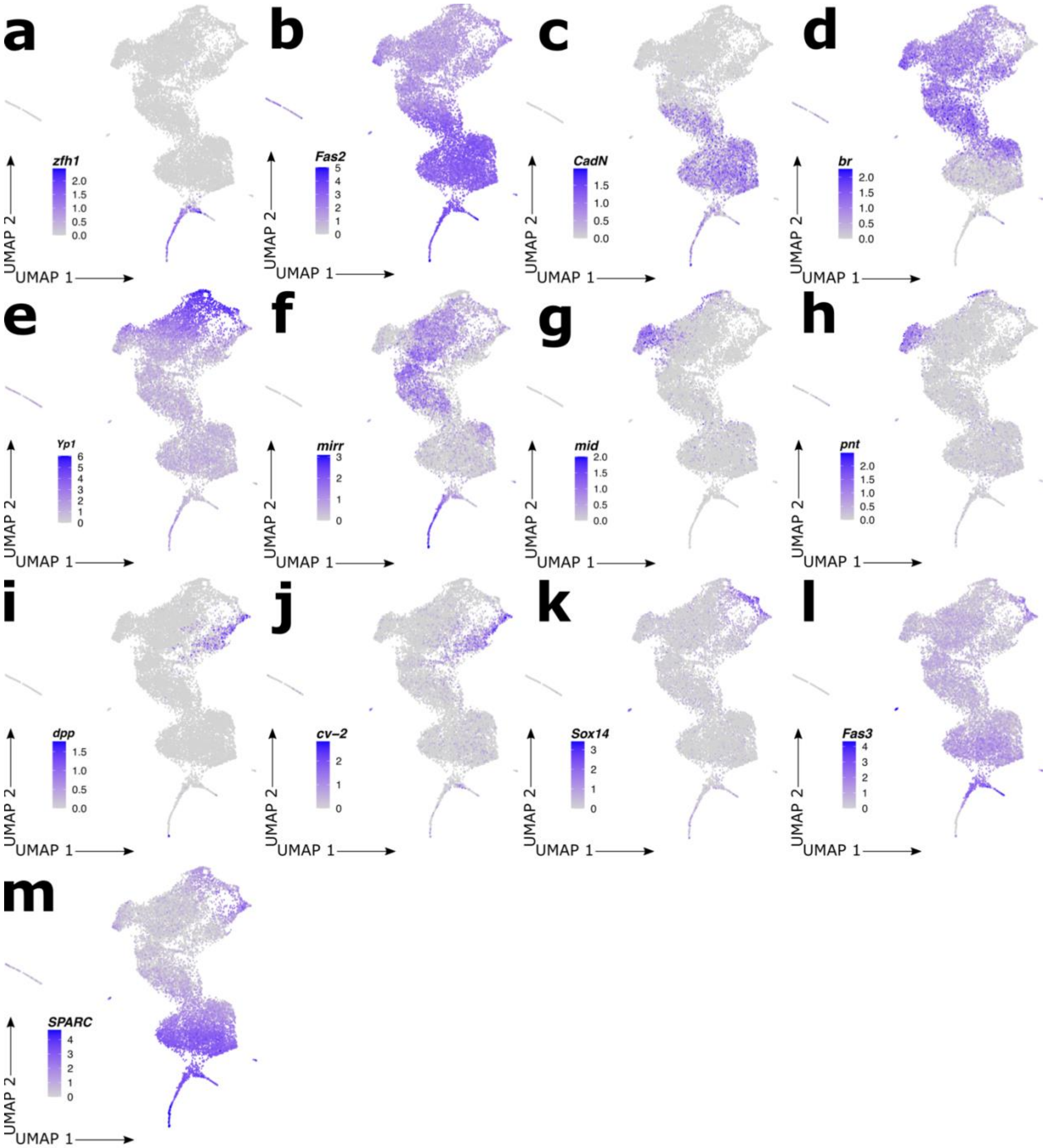

Supplementary Figure 7: Markers of main body cell populations and their derivatives

a-m) UMAP plots of selected markers as indicated.

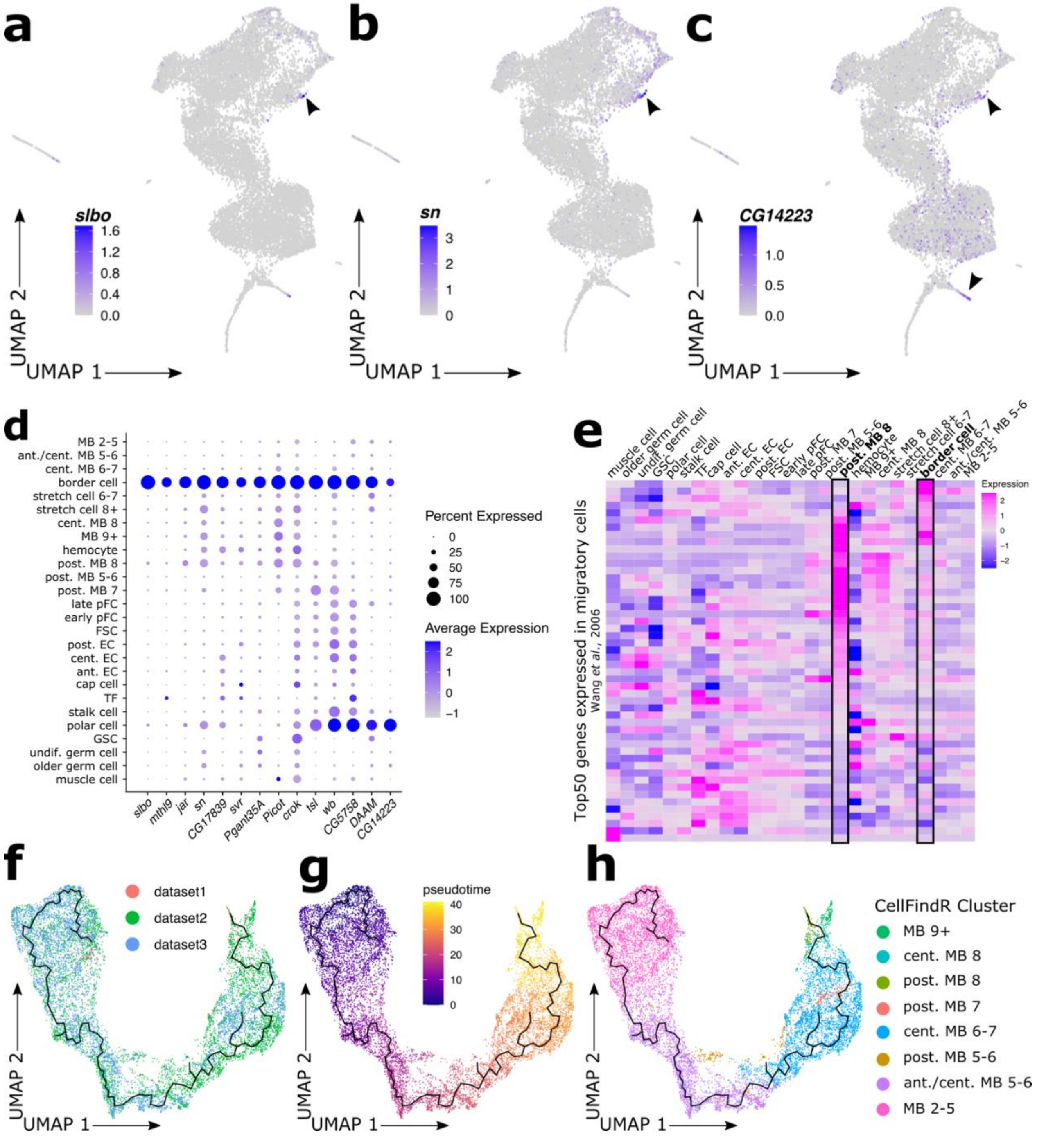

Supplementary Figure 8: Border cells and monocle3 analysis of main body follicle cells

a-d) UMAP plots (a-c) and a dot plot (d) showing the expression patterns of markers that are highly specific for the border cell cluster or for border cells and polar cells. Arrows in A-C point at clusters with high expression of the respective marker. e) Heat map showing the expression of the top 100 genes that are most strongly enriched in *slbo-Gal4*[7] expressing cells in each cluster. These genes are enriched in border cells and posterior follicle cells Stage 8 (boxes), as expected given the expression pattern of *slbo-Gal4*.[8] f-h) UMAP plots showing the pseudotime trajectory (black line) of main body follicle cells. The cells are

colored to display the distribution of each dataset across pseudotime (f); the temporal progression (g); and the position of cells in each cluster (h). undif.: undifferentiated; GSC: germline stem cell; TF: terminal filament; EC: escort cell; ant.: anterior; cent.: central; post.: posterior; FSC: follicle stem cell; pFC: prefollicle cell; MB: main body follicle cell.

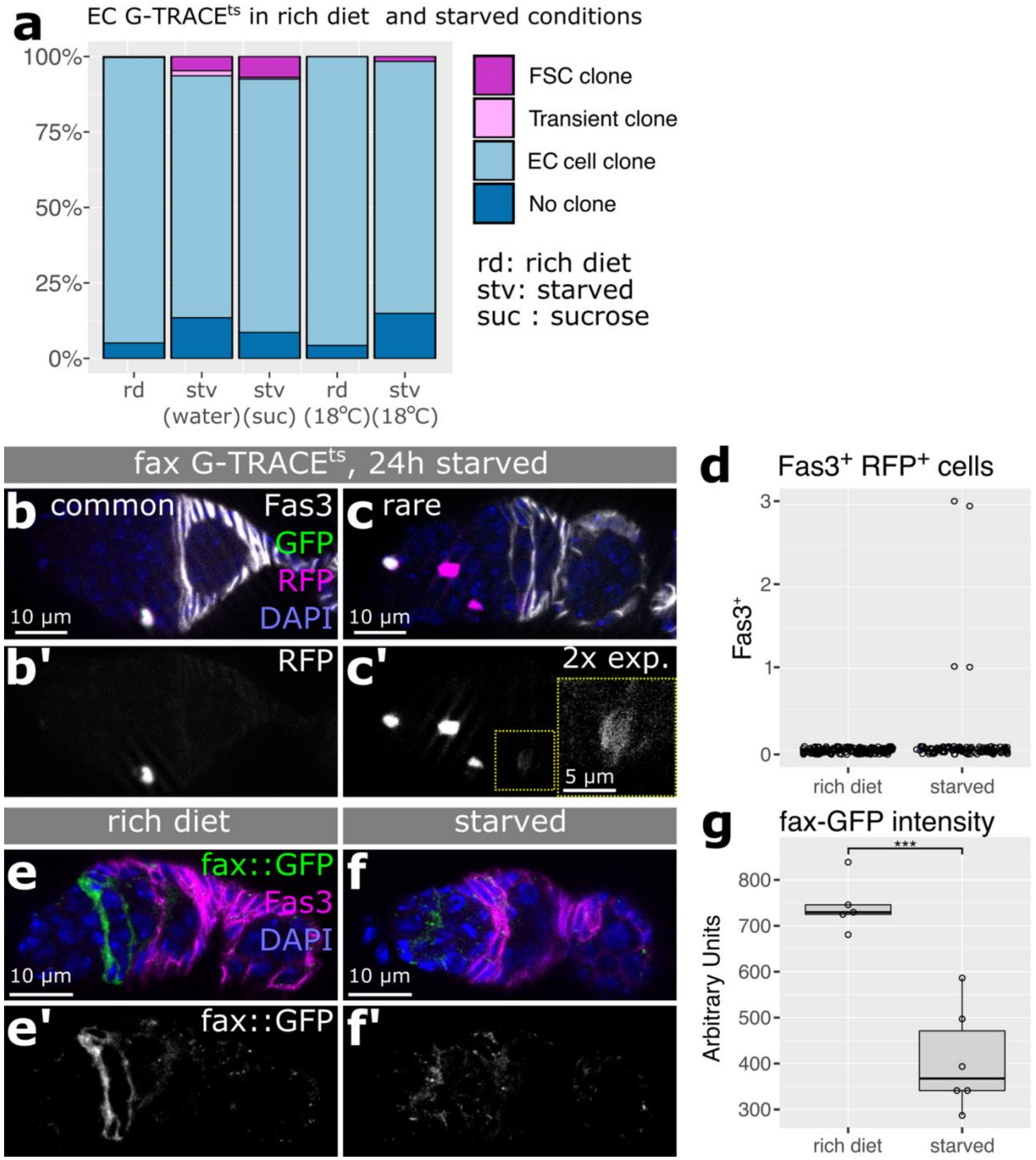

Supplementary Figure 9: EC conversion to FSCs and *fax* expression in response to nutrient deprivation

a) GFP^+ clone types in *fax-Gal4* driving G-TRACE^{ts} on rich diet, starved on water (29°C), protein starvation (sucrose, 29°C), or rich diet or water starvation at 18°C. Data for rich diet and starved (water) are combined with data in Fig. 8e, 9a. FSC and transient clones in both starvation conditions at 29°C confirms that nutrient deprivation induces ECs conversion to FSCs. Clones in total starvation at 18°C indicates that follicle cell

clones can form during starvation even when Gal4 is repressed by Gal80, indicating that follicle cell clones are not due to expression of *fax-Gal4* in follicle cells. However, fewer follicle cell clones are produced in this condition, suggesting that lower temperature partially suppresses the effect. p-values (two-sided Student's T-test) for comparisons between follicle cell clones (FSC + transient) in rich diet and starved (water): p = 0.01 or rich diet and starved (sucrose): p = 0.003. n = 374 (rich diet), 424 (starved (water)), 175 (starved (sucrose)), 164 (rich diet 18°C), 309 (starved 18°C) ovarioles. b-c) Germaria with *fax-Gal4* driving G-TRACE^{ts} dissected immediately following 24h exposure to indicated diet stained for DAPI (blue), GFP (green), RFP (magenta), and Fas3 (white). b'-c') RFP (white). b) Common expression pattern. c) Rarely observable RFP^{+} follicle cells with low signal. Inset: region of interest with twice the pixel intensity used for main image. d) Quantification of Fas3^{+} RFP^{+} cells. RFP in rare Fas3^{+} RFP^{+} cell (b) is very dim. n = 165 germaria (rich diet), 126 germaria (starvation). e-f) *fax::GFP* germaria under rich diet (d) and starvation (e) imaged with same settings and stained for DAPI (blue) and Fas3 (magenta). e'-f') fax::GFP (white). g) Quantification of fax-GFP intensity in germaria with rich diet or starvation. Intensity is significantly reduced upon starvation. n=5 germaria (rich diet), 6 germaria (starvation). ***p = 2 x 10^{-4} (two-sided Student's T-test). Box plots: midline corresponds to median; lower and upper hinges correspond to first and third quartiles; whiskers span smallest and largest values within 1.5 of interquartile range. EC: escort cell; FSC: follicle stem cell; rd: rich diet; stv: starved; suc: sucrose.

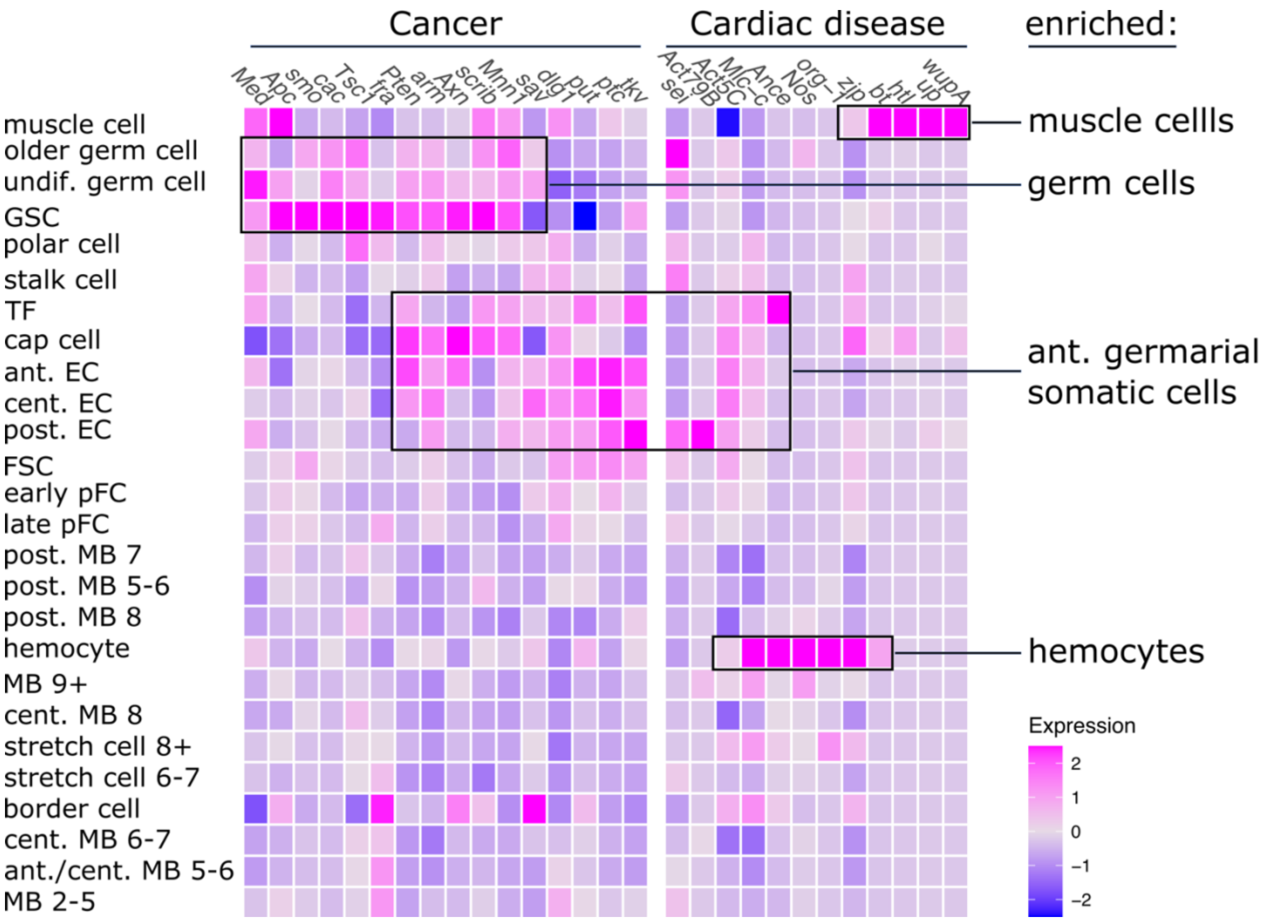

Supplementary Figure 10: Drosophila ovarian cell types with human disease gene expression profiles

A Heatmap showing enrichment for cells expressing cancer-associated genes in germ cell clusters, cancer and cardiac disease-associated genes in anterior germarial somatic cell clusters, and cardiac disease-associated genes in muscle cells and hemocytes. undif.: undifferentiated; GSC: germline stem cell; TF: terminal filament; EC: escort cell; ant.: anterior; cent.: central; post.: posterior; FSC: follicle stem cell; pFC: prefollicle cell; MB: main body follicle cell.

Supplementary Table 1: Table summarizing the number of high quality cells and the filter settings for each dataset.

	Dataset1	Dataset2	Dataset3	Total
High quality cells	435	8326	5412	14173
Filter settings				
nFeature	2000 - 6000	1000 - 3000	1800 - 4000	
nCount	5000 - 210000	1000 - 40000	2000 - 75000	

Supplementary Table 2: CellFindR clustering performance.

	Seurat Resolution						CFR	CFR + supervised subclusters
	0.2	0.5	2	3	5	10		
Clusters	7	14	28	40	56	91	22	26
marker	Number of clusters with marked cells							
Fas2	1	>1	>1	>1	>1	>1	1	1
upd1	<1	<1	<1	1	1	1	1	1
CG46339	<1	<1	<1	<1	<1	<1	1	1
Mhc	<1	<1	1	1	1	1	1	1
en	<1	<1	<1	<1	<1	<1	1	2

Comparison of CellFindR clustering results in combination with or without supervised sub-clustering based on markers identified with monocle3 with Seurat clustering results at 6 different resolution settings for several different cell types with well-defined markers. CFR: CellFindR.

Supplementary Table 3: Table listing the number of cells per cluster by dataset.

	Dataset1	Dataset2	Dataset3	Total
MB 2-5	160	2217	2161	4538
ant./cent. MB 5-6	56	1792	1360	3208
cent. MB 6-7	81	2609	922	3612
border cell	0	6	0	6
stretch cell 6-7	6	368	143	517
stretch cell 8+	16	267	76	359
cent. MB 8	16	177	38	231
MB 9+	11	164	24	199
hemocyte	5	23	0	28
post. MB 8	42	115	38	195
post. MB 5-6	4	130	75	339
post. MB 7	1	222	116	209
late pFC	4	54	64	122
early pFC	1	31	44	76
FSC	1	24	25	50
post. EC	6	26	31	64
cent. EC	0	4	74	78
ant. EC	1	1	32	34
cap cell	0	1	4	5
TF	1	0	10	11
stalk cell	3	15	36	54
polar cell	5	16	19	40
GSC	0	3	0	3
undif. germ cell	4	16	59	79
older germ cell	5	41	57	103
muscle cell	6	3	4	13

Supplementary References

1. Mével-Ninio, M., Fouilloux, E., Guénal, I. & Vincent, A. The three dominant female-sterile mutations of the Drosophila ovo gene are point mutations that create new translation-initiator AUG codons. Development 122, 4131–4138 (1996).
2. Vied, C., Reilein, A., Field, N. S. & Kalderon, D. Regulation of stem cells by intersecting gradients of long-range niche signals. Dev. Cell 23, 836–848 (2012).
3. Assa-Kunik, E., Torres, I., Schejter, E., Johnston, D. & Shilo, B. Drosophila follicle cells are patterned by multiple levels of Notch signaling and antagonism between the Notch and JAK/STAT pathways. Development 134, 1161–1169 (2007).
4. Wilcockson, S. G. & Ashe, H. L. Drosophila Ovarian Germline Stem Cell Cytocensor Projections Dynamically Receive and Attenuate BMP Signaling. Dev. Cell 50, 296–312.e5 (2019).
5. Tiwari, M. D., Zeitler, D. M., Meister, G. & Wodarz, A. Molecular profiling of stem cell-like female germ line cells in Drosophila delineates networks important for stemness and differentiation. Biol. Open 8, (2019).
6. Jia, D., Tamori, Y., Pyrowolakis, G. & Deng, W.-M. Regulation of broad by the Notch pathway affects timing of follicle cell development. Dev. Biol. 392, 52–61 (2014).
7. Wang, X. et al. Analysis of cell migration using whole-genome expression profiling of migratory cells in the Drosophila ovary. Dev. Cell 10, 483–495 (2006).
8. Rørth, P. Gal4 in the Drosophila female germline. Mech. Dev. 78, 113–118 (1998).
