# Supplementary material for: A single-cell atlas and lineage analysis of the adult Drosophila ovary
Source: Nat Commun. 2020 Nov 6;11:5628. doi: 10.1038/s41467-020-19361-0 (PMC7648648; doi:10.1038/s41467-020-19361-0)
Supplement: Supplementary file 15 — Description of Additional Supplementary Files [file 41467_2020_19361_MOESM15_ESM.pdf]

Title: Supplementary Data 1:
Description: DAVID 6.8 GO-term analysis of all cell types using marker genes identified with the CellFindR v2.0.0 get_stats command with $p < 0.01$.

Title: Supplementary Data 2:
Description: Summary of relevant GO-terms identified with DAVID 6.8 with explanation.

Title: Supplementary Data 3:
Description: CellFindR output listing the top 50 markers per cluster. This table was created using the CellFindR v2.0.0 get_maxtrix command.

Title: Supplementary Data 4:
Description: CellFindR output listing all differentially expressed genes. This table was created using the CellFindR v2.0.0 get_stats command.

Title: Supplementary Data 5:
Description: Seurat output listing all differentially expressed genes. This table was created using the Seurat v3.1.5 FindAllMarker command.

Title: Supplementary Data 6:
Description: Active regulons per cell type in dataset2. This table was created with SCENIC v1.1.2-2.

Title: Supplementary Data 7:
Description: Active regulons per cell type in dataset3. This table was created with SCENIC v1.1.2-2.

Title: Supplementary Data 8:
Description: Candidate genes changing expression in germ cell pseudotime identified by monocle3. This table was created using the monocle3 v0.2.1 graph_test command.

Title: Supplementary Data 9:
Description: Genes shared among EC subtypes. This table contains genes with p-value < 0.01 expressed in all EC subtypes and was created using the CellFindR v2.0.0 get_matrix command.

Title: Supplementary Data 10:
Description: Candidate genes changing expression in FSC and pFC pseudotime identified by monocle3. This table was created using the monocle3 v0.2.1 graph_test command.

Title: Supplementary Data 11:
Description: Candidate genes changing expression in main body follicle cell pseudotime identified by monocle3. This table was created using the monocle3 v0.2.1 graph_test command.

Title: Supplementary Data 12:

Description: Means and standard errors of the mean (S.E.M.) for all plots with stacked bars.

Title: Supplementary Software
Description:
File 1: R scripts used to produce plots and perform statistical analysis.
File 2: Seurat v3.1.5 script used for filtering and clustering.
File 3: monocle3 script used for pseudotime analysis.
File 4: SCENIC script used for analysis of gene regulatory networks
